# Evaluation of Online Consumer Reviews of Hospitals and Experiences of Racism Using Qualitative Methods

**DOI:** 10.1001/jamanetworkopen.2021.26118

**Published:** 2021-09-22

**Authors:** Jason Tong, Anietie U. Andy, Raina M. Merchant, Rachel R. Kelz

**Affiliations:** 1 National Clinician Scholars, Corporal Michael J. Crescenz Veterans Affairs Medical Center, Philadelphia, Pennsylvania; 2 Center for Digital Health, University of Pennsylvania, Philadelphia; 3 Department of Emergency Medicine, Perelman School of Medicine, University of Pennsylvania, Philadelphia; 4 Department of Surgery, Center for Surgery and Health Economics, Perelman School of Medicine, University of Pennsylvania, Philadelphia

## Abstract

This qualitative study examines online consumer reviews for experiences of racism in US hospitals among patients.

## Introduction

Racial disparities, the result of structural and interpersonal racism, represent a complex phenomenon present in all domains of health care.^1,2^ Although techniques to measure the quantitative impact of structural racism on racial disparities exist, the measurement of interpersonal racism is limited in health care because of its subjective nature. The inability to measure interpersonal racism at the local level has limited the ability to improve racist patient experiences within health care. Prior work has demonstrated consumer reviews’ unique ability to highlight novel concepts not captured in traditional performance metrics and to impact consumer hospital selection.^3^ Consumer reviews offer an opportunity to understand subjective perceptions of racism in health care in an unstructured and anonymous format. To demonstrate the potential role of consumer reviews in studying interpersonal racism in health care, we explored reviews of hospitals to better understand how consumers perceive and report racism.

## Methods

This qualitative study was deemed exempt by the University of Pennsylvania institutional review board and informed consent was not required because of the public and retrospective nature of the consumer reviews. This study followed the Strengthening the Standards for Reporting Qualitative Research (SRQR) reporting guideline.

A study of Yelp consumer reviews on United States hospitals published between January 2010 to January 2020 was performed. Yelp was selected because it is the most widely used referral website in the world, is frequently updated, and screens out potentially falsified reviews to prevent skewed ratings.^4^ Natural language processing was used to identify all reviews containing the terms “racist” or “racism.” All collected data were publicly available and no attempts at contacting reviewers or ascertaining reviewer gender, race, or ethnicity were made. Prior work has offered a conceptual framework delineating racism into distinct levels including institutional, interpersonal, and internalized.^5^ A random sample of reviews were analyzed to understand the content, followed by a formal qualitative content analysis to code recurrent themes and unique levels of racism. Episodes of racism were further classified into clinical and nonclinical environments. Content code queries were used to better understand the associations between identified codes.

## Results

During the study period, 90 786 online consumer reviews of US hospitals were obtained. Reviewer demographics were intentionally left anonymous for the purposes of this study. Of all reviews obtained, 260 reviews explicitly cited racism in 190 hospitals spread across 33 states. See sample reviews in Table 1. Among these, 179 reviews cited individual perpetrators of interpersonal racism, of which physicians (31% [56 of 179]) and nurses (53% [94 of 179]) were mentioned most. Interpersonal racism was associated with recurring themes including disrespect and unprofessionalism, incompetence, and acts of commission or omission. The most common themes included disrespect and unprofessionalism and acts of omission. See Table 2 for relative frequency of themes associated with each perpetrator category. There were 68 mentions of institutional racism, which were most frequently described as omitting standard care (26%; 18 of 68) and disrespectful (24%; 16 of 68). Episodes of racism were 2.5 times more frequently associated with clinical encounters (143 of 260) compared with nonclinical environments (58 of 260). Racism within nonclinical spaces occurred most commonly during interactions with receptionists and security guards. Nine reviews demonstrated internalized racist behavior by the consumer, primarily directed at nurses.

**Table 1.** Sample Quotes of Racist Experiences and Associated Coded Themes

Coded theme	Sample quote^a^^,^^b^
Domain	
Interpersonal	“Unprofessional employee at entrance… He is very rude. [And] racists.”
Institutional	“This hospital is RACIST against minorities and profiles women in pain!!”
Internalized	“The nurses are a bunch of hood kids who don’t give a half a **** about any patient. Don’t call me racist because you know it’s true.”
Setting	
Clinical	“And the Er doctor…Saw her and would not give her anything for pain…because she was Asian and my wife is Hispanic… that doctor is the biggest … Racist.”
Nonclinical	“Lady in release of information is racist and insensitive.”
Themes	
Unprofessional	“Dr… is the most racist unprofessional doctor I have ever met.”
Acts of omission	“Very rude and racist employees and rude doctors in this hospital… they did not help me they kicking me out of the hospital which is all about Discrimination against people from different countries.”
Incompetence	“… he is racist and disrespectful with the patient’s family and he discriminates people I was disappointed with his work ethic it was below average.”

^a^ Quotes are deidentified.

^b^ Quotes are unedited and may contain grammatical or spelling errors.

**Table 2.** Themes Associated With Interpersonal Racism by Identified Perpetrator Categories

Themes associated with interpersonal racism	Frequency of themes appearing in reviews, No./total No. (%)^a^
Nurses (94/179)^b^	
Stereotyping	8/94 (8.5)
Acts of commission	10/94 (10.6)
Incompetence	11/94 (11.7)
Acts of omission	21/94 (22.3)
Disrespect	33/94 (35.1)
Doctors (56/179)^b^	
Acts of commission	3/56 (5.4)
Stereotyping	5/56 (8.9)
Devaluation	8/56 (14.3)
Disrespect	11/56 (19.6)
Incompetence	11/56 (19.6)
Acts of omission	15/56 (26.8)
Reception (22/179)^b^	
Devaluation	2/22 (9.1)
Acts of omission	4/22 (18.2)
Disrespect	14/22 (63.6)
Security (13/179)^b^	
Devaluation	1/13 (7.7)
Stereotyping	1/13 (7.7)
Acts of commission	2/13 (15.4)
Disrespect	6/13 (46.2)

^a^ Percentages listed with themes reflect relative proportion of themes per individual perpetrator category.

^b^ Proportions listed with perpetrator categories reflect the number of reviews identifying these categories out of all reviews involving interpersonally mediated racism.

## Discussion

This qualitative study found that (1) it is feasible to identify acts of interpersonal racism in health care using qualitative methods on consumer reviews, (2) racism in health care can involve critical organization personnel outside of the clinical staff, and (3) racism can be bidirectional, affecting both patients and hospital employees. These detailed reviews likely represent the tip of the iceberg, and future efforts to supplement this data with existing hospital-based reporting measures, such as human resources reports, may help to further explore concepts such as patient-to-employee racism. Additionally, experiences of interpersonal racism are just one aspect of racism in health care, and future work is needed to couple the subjective measures of interpersonal racism with objective measures of structural racism to establish a hospital quality composite metric of racism. This study’s findings are limited by the low frequency of reviews citing racism, limited information on reviewer race, and additional context about reported experiences.
